# Efficient Deep Learning-Based Arrhythmia Detection Using Smartwatch ECG Electrocardiograms

**DOI:** 10.3390/s25175244

**Published:** 2025-08-23

**Authors:** Herwin Alayn Huillcen Baca, Flor de Luz Palomino Valdivia

**Affiliations:** Faculty of Engineering, Academic Department of Engineering and Information Technology, Jose Maria Arguedas National University, Andahuaylas 03701, Peru; fpalomino@unajma.edu.pe

**Keywords:** arrhythmia detection, deep learning, smartwatch, electrocardiogram, ECG, heart rate, CNN, cardiovascular diseases, PPG

## Abstract

According to the World Health Organization, cardiovascular diseases, including cardiac arrhythmias, are the leading cause of death worldwide due to their silent, asymptomatic nature. To address this problem, early and accurate diagnosis is crucial. Although this task is typically performed by a cardiologist, diagnosing arrhythmias can be imprecise due to the subjectivity of reading and interpreting electrocardiograms (ECGs), and electrocardiograms are often subject to noise and interference. Deep learning-based approaches present methods for automatically detecting arrhythmias and are positioned as an alternative to support cardiologists’ diagnoses. However, these methods are trained and tested only on open datasets of electrocardiograms from Holter devices, whose results aim to improve the accuracy of the state of the art, neglecting the efficiency of the model and its application in a practical clinical context. In this work, we propose an efficient model based on a 1D CNN architecture to detect arrhythmias from smartwatch ECGs, for subsequent deployment in a practical scenario for the monitoring and early detection of arrhythmias. Two datasets were used: UMass Medical School Simband for a binary arrhythmia detection model to evaluate its efficiency and effectiveness, and the MIT-BIH arrhythmia database to validate the multiclass model and compare it with state-of-the-art models. The results of the binary model achieved an accuracy of 64.81%, a sensitivity of 89.47%, and a specificity of 6.25%, demonstrating the model’s reliability, especially in specificity. Furthermore, the computational complexity was 1.2 million parameters and 68.48 MFlops, demonstrating the efficiency of the model. Finally, the results of the multiclass model achieved an accuracy of 99.57%, a sensitivity of 99.57%, and a specificity of 99.47%, making it one of the best state-of-the-art proposals and also reconfirming the reliability of the model.

## 1. Introduction

Cardiovascular diseases (CVDs) are the leading cause of death worldwide, accounting for 17.9 million deaths per year [1]. Among these diseases, arrhythmias are associated with cardiovascular events, heart failure, and sudden death [2]. Therefore, accurate diagnosis and early detection are crucial for receiving appropriate treatment, preventing subsequent complications, and even saving lives [3].

The standard diagnosis of an arrhythmia is performed by a cardiologist through the interpretation of an electrocardiogram (ECG). However, this diagnosis can be cumbersome and complex, leading to inaccuracies, as it is subject to the subjectivity of the cardiologist’s visual observation [4]. Additionally, the electrocardiograph is often affected by noise and interference, resulting in inaccurate ECGs and subsequent diagnostic errors [5]. Therefore, it is essential to provide reliable tools to support cardiologists in making accurate and timely diagnoses.

In recent years, deep learning techniques have achieved excellent results in automating arrhythmia diagnosis based on the information contained in ECG signals. Deep learning methods are continually refined, improving the accuracy and efficiency of arrhythmia detection [6].

Convolutional neural networks (CNNs) have been employed with great success in extracting local features from ECG signals and improving arrhythmia detection accuracy, offering the advantage of low-complexity techniques [5]. The state of the art also includes techniques that combine CNNs with others, such as LSTM and hybrid models [7,8,9], which improve accuracy but increase model complexity. Recently, Transformer models, with their attention mechanisms, have been proposed to enhance accuracy [5,6]. Despite their high computational complexity, they have achieved cutting-edge results. However, most deep learning-based proposals train and test their models on open ECG signal datasets from Holter devices, which limits the practical context of their results, especially for early arrhythmia detection.

Fortunately, technology has enabled the widespread use of smart wearable devices [10], such as smartwatches, which feature photoplethysmography (PPG) sensors that can record patients’ electrocardiograms (ECGs), making smartwatches a crucial tool for arrhythmia monitoring [11]. Numerous studies have proposed arrhythmia detection models based on ECGs from smartwatches [12,13,14,15,16,17], achieving good accuracy results. However, reliable arrhythmia detection results have not yet been achieved, nor are they oriented toward a practical clinical context for deploying the model on a smartwatch, as the focus is on accuracy, without considering that the model must be computationally lightweight.

In this context, we propose an efficient model for detecting arrhythmias based on ECG signals from smartwatches. The model is based on a one-dimensional (1D) convolutional neural network (CNN) architecture and utilizes two datasets: the UMass Medical School Simband dataset [18,19] for a binary arrhythmia detection model in smartwatches, to validate its efficiency and effectiveness, and the MIT-BIH arrhythmia database [20] for a multiclass arrhythmia detection model, to validate its results and compare them with the state of the art.

The model is designed for subsequent deployment on a smartwatch to monitor and detect arrhythmias as an early warning system, for which low computational complexity and FLOPS are crucial. It also aims to be a reliable model for arrhythmia detection, requiring cutting-edge results in accuracy, sensitivity, and specificity.

## 2. Related Work

Artificial intelligence-based ECG arrhythmia detection is a field of great interest to the scientific community, with numerous proposals and, above all, review articles published each year [21,22,23]. Therefore, related works are identified from these studies, as well as the most innovative proposals with cutting-edge results.

### 2.1. ECG Arrhythmia Detection in Classic Datasets

To analyze the results and methods of the most recent work, we used the MIT-BIH Arrhythmia dataset [20], a benchmark dataset that has become the standard for this type of work. The best and most recent proposals validate their methods on this dataset. In our work, we also use this dataset as a means of validating our proposal and comparing results with the state of the art.

Deep learning-based approaches are positioned as the most prominent and promising for detecting ECG arrhythmias. These models consist of single- or multi-layer structures that take ECG recordings as input. Each layer acts as a feature extractor, extracting the best classification or recognition patterns [24]. These approaches can be grouped according to the methods used, including works based on CNNs; combinations of CNNs, RNN, LSTM, and hybrids; and works based on Transformers [21].

Xia et al. [25] use a 1D CNN to detect atrial fibrillation. It utilizes the stationary wavelet transform (SWT) to convert the input into a 2D structure, which serves as input to the CNN. Ullah et al. [26] propose a 2D convolutional neural network (CNN) to classify ECG signals into eight beat classes. The 1D ECG signals are transformed into 2D spectrograms using the short-time Fourier transform (STFT). There is also the work of Zubair et al. [27] who present a temporal transition module with convolutional layers of different kernel sizes to capture short- and long-term patterns. They present a cost-sensitive loss function that adjusts class weights and solves the problem of data imbalance.

Some works modify CNN architecture with similar results. For instance, Rizqyawan et al. [28] propose a deep convolutional neural network (DNN) to classify arrhythmia using ECG signals without preprocessing. To address class imbalance, it employs a weighted loss function that adjusts the weights accordingly. Ojha et al. [29] present a 1D CNN model and an auto-encoder ACN to extract optimal features from ECG heartbeat windows; the extracted features are classified using SVM. Then, Jamil and Rahman [30] use a CNN with continuous wavelet transform (CWT)-based preprocessing to convert the signals into 2D signals for processing in the CNN. This approach also includes an attention block to extract a spatial feature vector. These approaches, utilizing a CNN architecture, have the advantage of low complexity and computational cost, in addition to achieving state-of-the-art results and being consolidated as the approach for our proposal.

Among the approaches that combine CNN and LSTM, there is the work of Chen et al. [7], who propose a model to classify six types of ECG signals in the MIT-BIH arrhythmia database. It employs a multi-input structure to process 10-second ECG segments, along with their corresponding RR intervals. Then, the work of Hassan et al. [8] proposes a deep learning model that combines a CNN with a bidirectional short-term and long-term memory network (BiLSTM) to classify five types of ECG signals. The model processes ECG signals without specifying additional preprocessing. Midani et al. [31] propose a deep learning model called “DeepArr,” which uses a sequential fusion method that combines deep feed-forward and recurrent neural networks to extract feature representations. In addition, Alamatsaz et al. [9] propose a deep learning model based on a convolutional neural network (CNN) with an LSTM attention block to classify five types of arrhythmias. ECG signals are transformed into 2D images using a continuous wavelet transform (CWT), allowing for the capture of time–frequency features. These approaches yield similar results to CNNs, but their computational complexity and costs are higher due to the use of LSTM.

We cannot fail to mention Transformer-based works. They are more recent but not necessarily state of the art. For example, Akan et al. [32] present a Transformer architecture for classifying arrhythmias in ECG signals. The technique uses multiple multi-head attention layers to capture complex temporal and spatial relationships in ECG data, along with positional encoding to maintain the sequential order of the signals. Another study is presented by Islam et al. [4], who propose CAT-Net, a model that combines multi-head attention layers and a Transformer encoder to classify arrhythmias. The convolutional layers capture local morphological features of the heartbeats, while multi-head attention and the Transformer extract global contextual information.

El-Ghaish et al. [6] propose ECGTransForm, a Bidirectional Transformer (BiTrans)-based model that captures temporal dependencies of previous and subsequent contexts and integrates multi-scale convolutions to extract spatial features at different granularities, and a channel recalibration module to improve feature salience. In addition, the work of Kim et al. [5] uses the Stockwell transform to convert time-to-frequency signals and extract features. A CNN captures local patterns, while a Transformer architecture models long-term dependencies without requiring peak detection. Table 1 presents a summary of the results of the different works tested on the MIT-BIH Arrhythmia dataset [20].

Recently, TinyML and Transformer-lite architectures optimized for wearable devices have been developed, aiming to achieve high accuracy while maintaining minimal computation and memory requirements. Busia et al. [33] propose a tiny Transformer for ECG classification on the MIT-BIH arrhythmia database, with 98.97% accuracy, optimized for wearables, achieving 4.28 ms of inference and 0.09 mJ on the GAP9 processor. Kim et al. [34] propose TinyCES, a TinyML-based ECG classification system that processes data directly on the device, using CNNs on the MIT-BIH arrhythmia database and PTB Diagnostic ECG Database, achieving 97% accuracy on an Arduino prototype. Alvarado et al. [35] developed a TinyML-based wearable cardiac monitoring system to detect arrhythmias in real time using the PTB-XL dataset, achieving 95% accuracy and an 88% reduction in false positives.

These works demonstrate the potential of combining wearable efficiency with optimization for detecting and monitoring arrhythmias. By contrast, our CNN-based approach focuses on maximizing classification performance while maintaining an efficient Conv1D architecture and allows for future adaptation for deployment on wearable platforms.

### 2.2. ECG Arrhythmia Detection in Smartwatches

Recently, there have been significant technological advances in wearable devices, especially smartwatches, for arrhythmia detection. Despite being consumer-grade devices, advanced smartwatches can measure health data comparable to electrocardiographs. A notable advantage of these devices is their ability to provide continuous, noninvasive monitoring. These features are crucial for identifying asymptomatic arrhythmias and achieving the early detection of arrhythmias [36].

Smartwatches utilize photoplethysmography (PPG) technology to detect irregularities that may indicate underlying arrhythmias. PPG is a noninvasive technology for monitoring heart rate by detecting changes in blood volume through the skin. The device uses a light source directed at the skin. The light penetrates the skin and reflects off blood vessels. A photodetector measures the amount of reflected light, which varies with blood volume with each heartbeat [37].

We compiled the most recent studies seeking to detect ECG arrhythmias using smartwatches. We searched for reviews and some more recent studies [38,39,40]. We carefully selected those with measurable results, recent ones, and, above all, those based on smartwatches. These studies serve as a benchmark against which to compare the results of our proposal.

Avran et al. [12] evaluated atrial fibrillation (AF) detection using an Apple Watch Series 4 smartwatch in 204 patients, comparing its performance with a 12-lead Holter monitor. They used a machine learning-based algorithm to identify AF. The results showed a sensitivity of 88% and a specificity of 98%. Subsequently, Ploux et al. [13] evaluated atrial fibrillation (AF) detection using an Apple Watch Series 4 smartwatch in 260 patients. A deep neural network-based algorithm was used to analyze PPG signals and classify AF. The results reported an accuracy of 92%, a sensitivity of 91%, and a specificity of 94%.

We also identified the work of Ford et al. [14], who utilized an Apple Watch Series 4 smartwatch to detect arrhythmias using PPG signals and compare the results with those from an electrocardiograph. They utilized a machine learning algorithm, achieving an accuracy of 87%, a sensitivity of 68%, and a specificity of 93%. A study with 200 patients by Abu-Alrub et al. [15] used a Samsung Galaxy Watch 3, achieving 88% sensitivity and 81% specificity. This research highlights the usability of the smartwatch for the continuous monitoring of arrhythmia episodes.

More recent work with cutting-edge results is found in Wasserlauf et al. [16], who treated 250 patients using an Apple Watch 5. The approach utilized deep learning to process and classify arrhythmias, achieving a sensitivity of 25% and a specificity of 99%. Its low sensitivity indicated limitations in detecting false positives. Mannhart et al. [17] presentaron otro estudio, en el que probaron varios modelos de relojes inteligentes comerciales. Utilizando los resultados del Galaxy Watch 4 en 201 pacientes, los resultados no fueron óptimos, con una sensibilidad del 58% y una especificidad del 75%. El trabajo sugiere que los profesionales sanitarios deberían diagnosticar posteriormente cualquier problema de salud detectado mediante relojes inteligentes. Table 2 presents a summary of the results of the aforementioned works.

## 3. Materials and Methods

- Datasets: UMass Medical School Simband Dataset [18,19], and MIT-BIH Arrhythmia Database [20].
- Anaconda Navigator (Version 2.6.3).
- Jupyter Notebook (version 7.3.2).
- Python (Version 3.12.19).
- Tensorflow (Version: 2.19.0).
- Keras (Version: 3.10.0).
- Pandas (Version: 2.2.3).
- Scikit-learn (Version: 1.7.1).
- Numpy (Version: 2.0.1).
- PyWavelets (Version: 1.8.0).
- Matplotlib (Version: 3.10.1).
- Scipy (Version: 1.15.2).

### 3.1. Proposed Model

A model is proposed for detecting arrhythmias from electrocardiogram (ECG) data from smartwatches. When deployed on a smartwatch, the model monitors and displays early warnings about the possible detection of arrhythmias. Therefore, given the low computational power of smartwatches, the deployed model must be lightweight; that is, the model architecture should be simple and efficient.

**Figure 1:**
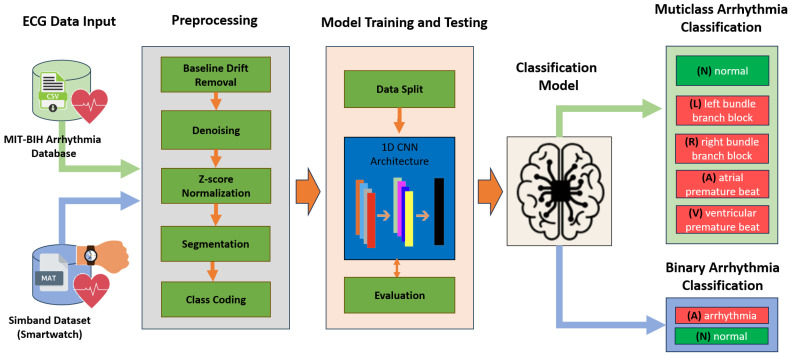
The pipeline of the proposed model.

Under this approach, we propose an architecture based on a one-dimensional neural network (1D CNN), which takes electrocardiogram data as input, performs multi-stage preprocessing, trains and tests the model to extract features, evaluates the model by extracting metrics, and finally validates the model by detecting arrhythmias. Figure 1 shows the pipeline of the proposed model.

### 3.2. ECG Data Input

Two datasets were used as input.

#### 3.2.1. MIT-BIH Arrhythmia Database

The dataset corresponds to electrocardiogram (ECG) signals from Holter devices, compiled by the Massachusetts Institute of Technology (MIT) and Beth Israel Deaconess Medical Center in Boston [20]. It includes ECG recordings from 118 normal patients and 48 patients with arrhythmias (N: normal, L: left bundle branch block, R: right bundle branch block, A: atrial premature beat, V: ventricular premature beat). The ECG signals were recorded at a sampling rate of 128 Hz using an Ampex VR-66/IVA digital tape recorder. It records twelve 30 min ECG recordings at a sampling rate of 128 Hz.

Although these electrocardiogram data do not come from smartwatches but from Holter monitors, we used this dataset as a basis to validate the reliability of our model and compare our results with the state of the art, as it is the reference dataset used by the most innovative proposals.

#### 3.2.2. UMass Medical School Simband Dataset

Data were taken from 37 patients (28 men and 9 women) with and without cardiac arrhythmia, ranging in age from 50 to 91 years. Patients simultaneously wore smartwatches and Holter monitors and were monitored in the outpatient cardiovascular clinic at the University of Massachusetts Medical Center (UMMC) [18,19]. This dataset contains data from normal (NSR) patients and patients with arrhythmias, including atrial fibrillation (AF), premature atrial contractions (PACs), and premature ventricular contractions (PVCs). ECG and smartwatch data were measured simultaneously on the chest and wrist using a 7-lead Holter monitor and a Samsung Simband 2 smartwatch. The smartwatch data consisted of 8-channel PPG signals, three-axis accelerometers, and a single-lead ECG. The PPG signals from the smartwatch were sampled at 128 Hz. All signals were segmented into 30 s lengths. Single-lead ECG data were taken for our study.

Although the data are divided into arrhythmia classes (NSR, AF, PAC, and VS), data specifically corresponding to smartwatch ECG recordings were not present in all patients, nor were there recordings of the specific arrhythmia type. Therefore, arrhythmia classes were grouped into two classes, making our model binary in detection: (N) normal; (A) arrhythmia. We used this dataset as the basis for our results. Access had to be requested through requests to the authors (https://github.com/Cassey2016/UMass_Simband_Dataset, accessed on 13 July 2025), as, to our knowledge, there is no publicly available ECG dataset from smartwatches.

### 3.3. Preprocessing

The following steps were used when processing both datasets.

#### 3.3.1. Baseline Drift Removal

A specific procedure was implemented to eliminate baseline drift, which is primarily due to low-frequency artifacts generated by the patient’s breathing or movement. This distortion can affect the correct identification of ECG signals and the model’s classification results. To correct this, a digital high-pass filter with a cutoff frequency of 0.5 Hz was applied, designed using a 4th-order Butterworth filter. This filter attenuates very low-frequency components, preserving the morphology of the ECG signal. The process was performed on each signal segment before applying the subsequent steps of denoising, z-score normalization, and segmentation.

#### 3.3.2. Denoising

Denoising is crucial in the preprocessing of electrocardiograms (ECGs), particularly in smartwatch signals, such as the Simband dataset, where noise can amplify detection errors. The goal is to minimize artifacts that could distort heartbeat morphologies, thereby enhancing segmentation accuracy and feature extraction for the CNN.

**Figure 2:**
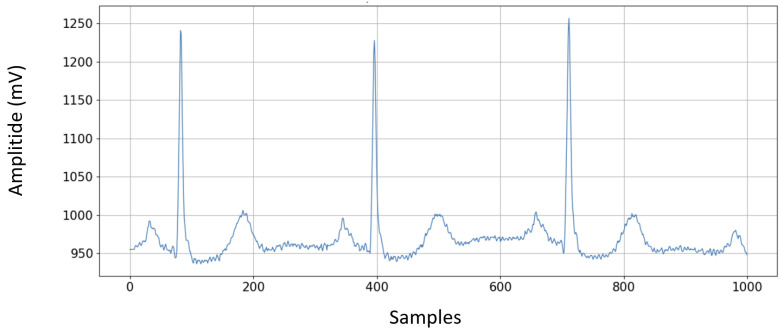
ECG signals from a random patient from the MIT-BIH arrhythmia database in a window of 1000 samples.

A discrete wavelet transform (sym4) was applied to each window of 1000 samples extracted from the ECG segments (see Figure 2). The signal is decomposed to the maximum allowable level, and the detail coefficients are filtered with a fixed threshold (0.04 times the maximum value of each level). ECG signals were obtained from a random patient from the MIT-BIH arrhythmia database in a window of 1000 samples.

#### 3.3.3. Z-Score Normalization

This stage standardizes the voltage values to have a mean of 0 and a standard deviation of 1. This is a common practice in CNN preprocessing. The idea is to standardize the amplitudes so that the CNN processes consistent signals, regardless of variations in the voltage scale, such as those caused by the use of different equipment. This improves training convergence.

#### 3.3.4. Segmentation

Each patient’s ECG recordings were divided into sets of 1000 records, called segments, which serve as input for the CNN, ensuring that each segment contains complete beats in a temporal context. The idea is to ensure that R peaks are captured in each segment and that the relevant morphologies are obtained.

Each ECG segment is divided into windows of 1000 samples, each approximately 7.81 s long, since the sampling frequency in both datasets is 128 Hz. Windows are extracted if they have exactly 1000 samples and are not labeled as noise.

#### 3.3.5. Class Coding

Specific labels are assigned to each segment, allowing the model to learn specific patterns for each class. One-hot encoding is used, which is compatible with the categorical loss used in training.

In the MIT-BIH arrhythmia database [20], each segment is labeled according to the spike class R (N, L, R, A, and V) obtained from the class annotations. The labels are converted to one-hot format (vectors of length 5). In the Simband dataset [18,19], each segment initially has a label (0.0 for NSR, 1.0 for AF, 2.0 for PAC, and 3.0 for PVC). Labels 1.0, 2.0, and 3.0 are grouped as Arrhythmia (binary label 1), and 0.0 as Normal (label 0). The labels are encoded in one-hot format (vectors of length 2).

### 3.4. Model Training and Testing

#### 3.4.1. Data Split

In both datasets, the segments with 1000-sample windows and their one-hot labels were divided into training and test sets, with 80% allocated to training and 20% to testing. To achieve this, a patient-level partition was performed instead of the classic random partitioning, ensuring that

- Each patient was uniquely included in either the training set or the test set.
- All ECG windows from the same patient remained in the same set.

Therefore, we applied a Leave-One-Subject-Out Cross-Validation (LOSO) validation scheme, in which each patient is excluded from the training set and used as a test set in a separate iteration. This approach eliminates the possibility of information leakage between sets and allows for a more realistic assessment of the model’s ability to generalize to new patients.

#### 3.4.2. Model Architecture

The model architecture for multiclass arrhythmia detection in the MIT-BIH arrhythmia database [20] is based on a 1D CNN, which receives preprocessed windows of 1000 samples. The network consists of four convolutional layers (Conv1D) with 16, 32, 64, and 128 filters, and kernel sizes of 11, 13, 15, and 17, respectively. All layers use ReLU activation and the ‘same’ padding to maintain the input length. Each convolutional layer is followed by a max pooling layer (pool size 2), progressively reducing the length from 1000 to 999, 499, 249, and 124 samples, extracting temporal features.

**Figure 3:**
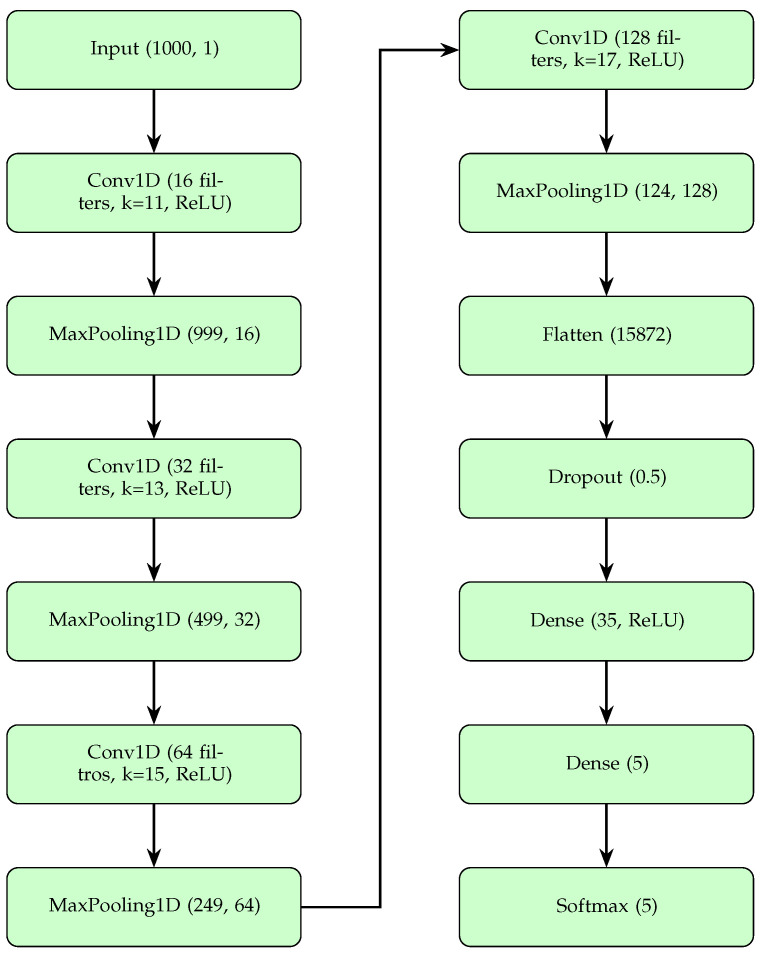
CNN architecture for MIT-BIH arrhythmia database (4-layer 1D CNN).

After the convolutional layers, the output of (124, 128) is flattened into a 15,872-element vector using a Flatten layer, followed by a Dropout layer to eliminate overfitting. Then, a dense layer with 35 neurons (ReLU) combines the extracted features, and a final dense layer with five neurons combines classes N, L, R, A, and V. A softmax layer converts these logits into probabilities, achieving multiclass classification. Figure 3 graphically shows this architecture.

**Figure 4:**
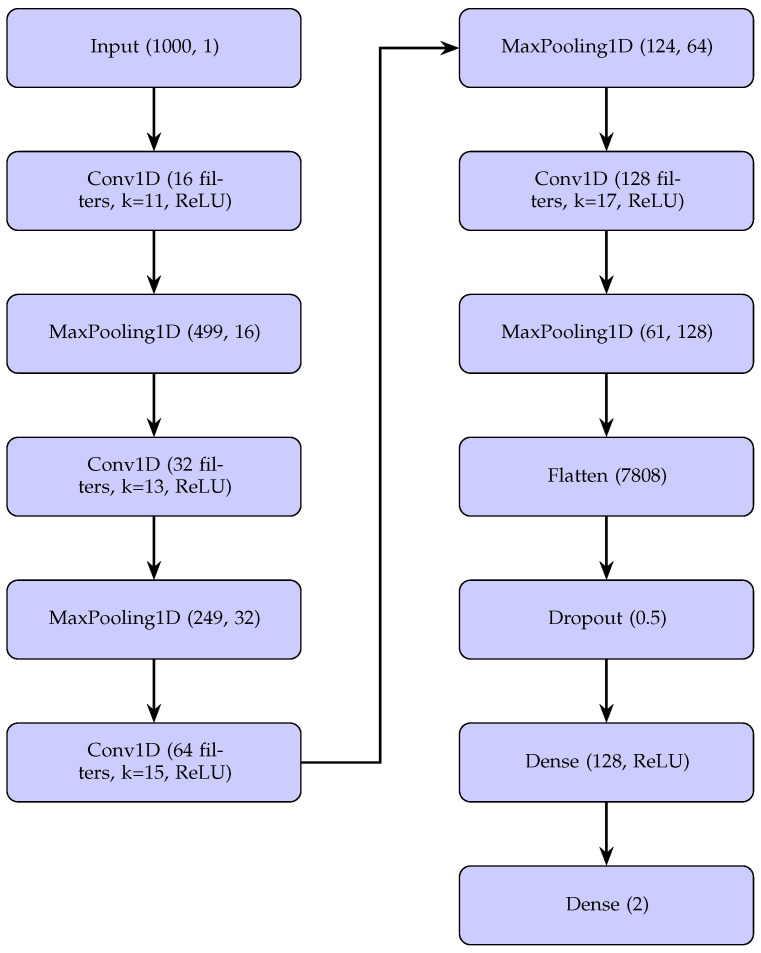
CNN architecture for UMass Medical School Simband dataset (4-layer 1D CNN).

The model architecture for arrhythmia detection on the UMass Medical School Simband dataset [18,19] is also based on a 1D CNN, with an input of a 1000-sample segment. The network consists of four convolutional layers (Conv1D) followed by MaxPooling1D layers, each with filters of 16, 32, 64, and 128. The first convolutional layer applies 16 filters with a kernel size of 13, followed by a MaxPooling1D layer that reduces the length from 1000 to 499. The following layers use kernels of sizes 11, 13, 15, and 17, with 32, 64, and 128 filters, respectively, and each is followed by a MaxPooling1D that reduces the dimensions to 249, 124, and 61. The output is then flattened into a 7808-element vector by a Flatten layer, followed by a Dropout layer. Finally, two dense layers are used: the first with 128 units and ReLU activation, and the second with 2 units for a binary classification of (N) normal and (A) arrhythmia. Figure 4 graphically shows this architecture.

Although both architectures share a similar structure, comprising four Conv1D and four MaxPooling1D, Flatten, Dropout, and Dense layers, with kernel sizes of 11, 13, 15, and 17 and numbers of filters of 16, 32, 64, and 128, the architecture for the Simband dataset [18,19] presents several differences. First, the number of output classes is two versus five. Furthermore, the intermediate dense layer in Simband has 128 units, compared to 35. Another difference is the output of the Flatten layer. In Simband, it produces a vector of 7,808 elements because the last MaxPooling1D reduces the dimension to 61 with 128 filters, while in MIT-BIH, it produces 15,872 elements.

## 4. Experiments and Results

The proposal was implemented and evaluated using Python version 3.12.9, as a Jupyter notebook on the Anaconda platform. A GPU was not required, as the model’s low complexity meant training only took 20 to 30 minutes in both datasets. The following training parameters were used:

- Epochs: 50;
- Batch size: 32;
- Loss function: Categorical cross-entropy;
- Optimizer: Adam.

After training and generating the arrhythmia detection model, the model was evaluated using the following metrics:

- Accuracy: Measures the model’s precision, i.e., the proportion of correct predictions out of the total samples.
- Sensitivity: Measures the proportion of positive cases correctly classified per class.
- Specificity: Measures the proportion of negative cases correctly identified per class.
- F1-score: Measures the proportion of how well it correctly identifies positives without generating many false positives or negatives.
- AUROC: Measures the ability of a classification model to distinguish between classes.

### 4.1. Results in UMass Medical School Simband Dataset [18,19]—Effectiveness and Efficiency

The model converged in the first 20 epochs, achieving the following effectiveness global results:

- Accuracy: 64.81%;
- Sensitivity: 89.47%;
- Specificity: 6.25%;
- F1-score: 78.16%;
- AUROC: 0.1978;
- AUROC 95% bootstrap CI: 0.1446–0.2485;
- F1-score 95% bootstrap CI: 0.7445–0.8189.

**Figure 5:**
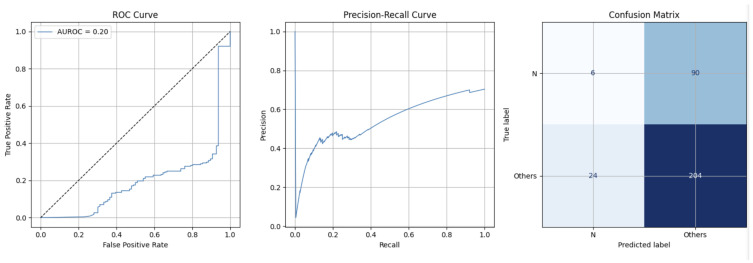
ROC Curve, Precision–Recall Curve, and Confusion Matrix in UMass Medical School Simband dataset.

To calculate the efficiency of the binary model, the computational complexity was measured, reaching 1.2 million parameters and 68.48 MFlops. Additionally, Table 3 shows the results of accuracy, sensitivity, and specificity by class. Finally, Figure 5 shows the ROC Curve, Precision–Recall Curve, and Confusion Matrix.

### 4.2. Results in MIT-BIH Arrhythmia Database [20]

**Figure 6:**
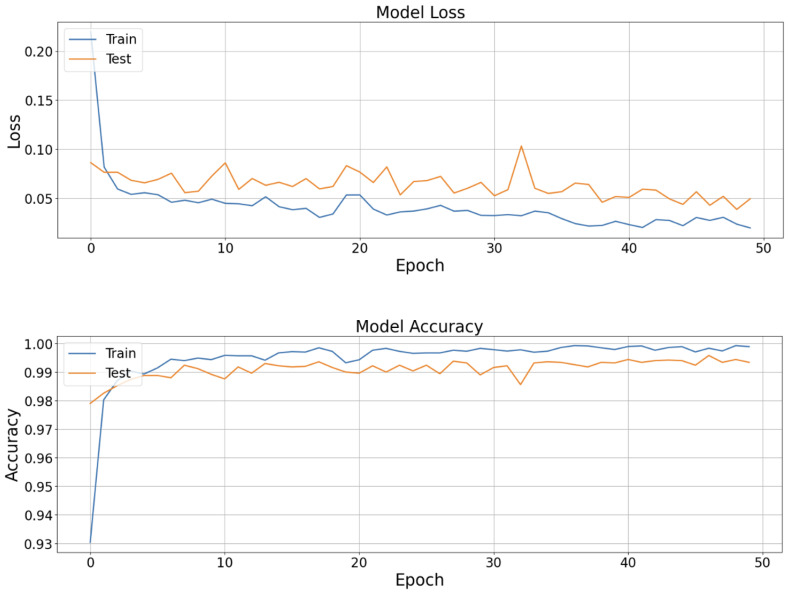
Loss and accuracy history in the training model.

In this dataset, convergence was also short. Figure 6 shows that the model stabilizes within the first 15 epochs, after which it achieves good results.

- Accuracy: 99.57%;
- Sensitivity: 99.57%;
- Specificity: 99.47%;
- F1-score: 0.9957;
- AUROC: 0.9958;
- AUROC 95% bootstrap CI: 0.9943–0.9972;
- F1-score 95% bootstrap CI: 0.9946–0.9966.

**Figure 7:**
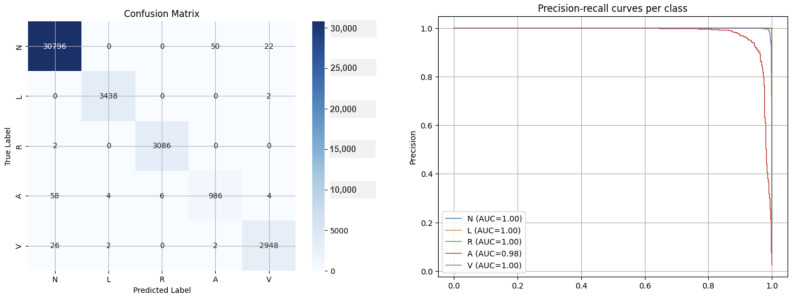
Confusion Matrix and Precision–Recall Curve per class in MIT-BIH arrhythmia database.

Additionally, Table 4 shows the results of accuracy, sensitivity, and specificity by class (N, L, R, A, and V). Finally, Figure 7 shows the Precision–Recall Curve and Confusion Matrix.

## 5. Discussion

### 5.1. Discussion of Results from UMass Medical School Simband Dataset [18,19]—Effectiveness and Efficiency

The overall results of our proposal on the Simband dataset are acceptable, achieving an overall accuracy of 64.81%. This result suggests that our model could be deployed on smartwatches.

In a clinical setting, sensitivity, or true positives, is the most relevant metric, as it is important to understand how well it detects patients with arrhythmia. Our model achieved 89.47%. This is a crucial result for the objectives of our work, as it confirms that our model is effective in detecting arrhythmias when processing ECGs from smartwatches. On the other hand, the specificity results, or how well it detects patients without arrhythmia, reached 6.25%. This result suggests that our model is also ineffective in detecting patients without arrhythmia.

It should be noted that these results respond to the specific characteristics of electrocardiograms (ECGs) from Samsung’s Simband 2 smartwatches only, which raises the possibility of overfitting and model specialization. Therefore, generalizing the model to ECGs from other smartwatches remains unproven. It would be necessary to test it with such data. Unfortunately, to our knowledge, there are no open ECG data from smartwatches of other models and brands.

However, considering the small size of the Simband dataset (37 patients), to avoid overfitting, we used data partitioning using a Leave-One-Subject-Out Cross-Validation (LOSO) scheme, ensuring that each patient is only in the training or test set, and ECG windows from the same patient remain in the same set.

This scheme better simulates a real-life clinical application, where the model must detect arrhythmias in patients not seen during training. This eliminates the possibility of information leakage between sets and allows for a more realistic evaluation of the model for generalization to new patients. Furthermore, the Simband dataset is imbalanced (29.63% Normal and 73.7% Arrhythmia), and using resampling affects the minority class and skews the results (as analyzed in sect:Ablation Study). Therefore, our proposal processes the data without resampling, yielding more realistic and unbiased results.

On the other hand, our model is binary on the Simband dataset, meaning it only has two classes: (N) Normal and (A) Arrhythmia. This fact could undermine our model’s merit. However, in a subsequent deployment of our proposal in a smartwatch app, the detection of specific arrhythmia classes is not yet reliable, as the reliability of smartwatch PPG technology is not yet comparable to that of electrocardiographs or Holter devices. Therefore, it is more reliable to detect only arrhythmia in general and subsequently diagnose, classify, and treat it using healthcare professionals.

To further validate our model, our results were compared with the most important and innovative state-of-the-art proposals (see Table 5). Our accuracy, sensitivity, and specificity results, 68.41%, 89.47%, and 6.25%, respectively, are below those of most other studies. However, our proposal almost surpasses the state of the art in sensitivity (89.47%) and confirms that our model is effective in detecting arrhythmias when processing ECGs from smartwatches.

Regarding efficiency results, computational complexity was measured, reaching 1.2 million parameters and 68.48 MFlops. To our knowledge, there are no review articles on the efficiency of models for detecting arrhythmias in electrocardiogram data, nor did we find efficiency results in related studies, and we have no references against which to compare the results. However, we can demonstrate that our 1D CNN architecture is more efficient considering both the nature of the data and the computational complexity and cost:

- ECG signals are one-dimensional time series. One-dimensional CNNs directly process this structure, avoiding conversions to two-dimensional representations that involve more preprocessing, storage, and the potential loss of fine-grained temporal information.
- Lower parameter count. A typical 1D CNN for ECG (four Conv1D layers with small filters) typically has between $10^{4}$ and $10^{5}$ parameters, while 2D-CNN, LSTM, or Transformer-based architectures for the same task can easily exceed $10^{6}$ parameters.
- Lower FLOPs. Such a 1D CNN can require on the order of $10^{6}$ to $10^{7}$ FLOPs, compared to $10^{8}$ or more for equivalent 2D or LSTM/Transformer networks, resulting in faster inference times.
- Suitability for resource-constrained devices. The lower number of parameters and FLOPs reduces memory usage and allows execution on microcontrollers, embedded hardware, or smartwatches without sacrificing accuracy.

### 5.2. Discussion of Results from MIT-BIH Arrhythmia Database [20]

The evaluation and comparison of our binary model with state-of-the-art proposals lack a standard dataset for equal comparisons. MIT-BIH is the benchmark dataset for multiclass arrhythmia detection. There are many results from proposals using this dataset. For this reason, we chose to use this dataset to test our model in a multiclass scenario and compare its results with the state-of-the-art datasets. This allows us to validate our proposal’s architecture, even though the data are not from smartwatches but from Holter devices. Our overall accuracy, sensitivity, and specificity results—99.57%, 99.57%, and 99.47%, respectively—place us among the best state-of-the-art proposals (see Table 6). These results confirm that our model is reliable and can also be used as a benchmark for detecting multiclass arrhythmias with Holter device data. Another of our goals was to make our model lightweight in terms of complexity. As can be seen in Table 6, the most recent proposals use Transformer-Attention as their primary method. These proposals have high computational complexity and also fail to outperform our method. The same is true for proposals that combine CNN and LSTM. Thus, our 1D CNN-based model achieves the best results and the lowest complexity.

## 6. Ablation Study

### 6.1. An Ablation Study of the Model Tested on the UMass Medical School Simband Dataset [18,19]—Effectiveness and Efficiency

As clarified in sect:Discussion, our proposed model uses the Leave-One-Subject-Out Cross-Validation (LOSO) validation technique and does not resample the dataset, thus achieving accuracy, sensitivity, specificity, and AUROC values of 64.81, 89.47, 6.25, and 62.5%, respectively.

However, resampling the data and ensuring that each class (Normal and Arrhythmia) accounts for 50% of the variance improve the metrics, achieving values of 66.05, 98.72, 52.1, and 65.8%, respectively. Given the highly imbalanced Simband dataset (29.63% Normal and 70.37% Arrhythmia), this variant is not realistic, as it affects the metrics of the less dominant class.

Furthermore, if Leave-One-Subject-Out Cross-Validation (LOSO) is eliminated after resampling, replacing it with a random 80/20 data partition, excellent results of 98.73, 98.73, 97.52, and 97.7% are achieved. These results are almost perfect and surpass the state of the art (see Table 7), but this variant is also unrealistic. It can introduce data from the same patient in both training and validation, artificially inflating model performance due to the morphological specificity of the ECG between subjects. Therefore, it is discarded.

Tests were also run by reducing the four convolutional layers to three, and the metrics decreased considerably. Finally, the kernel sizes of the four convolutional layers (11, 13, 15, 17) were incremented to (13, 15, 17, 19), and the metrics also decreased (see Table 7).

**Figure 8:**
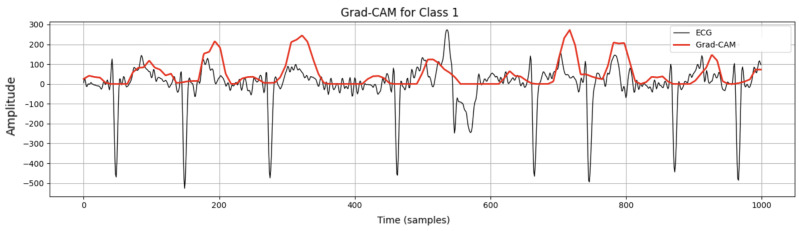
Grad-CAM in the first input sample on the Simband dataset.

To determine which regions of the input ECG signals were most important for our 1D CNN model to make a decision, the Grad-CAM explanation technique was applied to the first input sample (see Figure 8). Since a typical smartwatch signal is noisy and highly variable, the exact activation segment cannot be identified. However, it can be seen that it involves the R peak and the PQR segments. Therefore, the PQR segment is the most important region for arrhythmia detection in smartwatches.

### 6.2. An Ablation Study of the Model Tested on the MIT-BIH Arrhythmia Database [20]

As with our binary model, the proposed multiclass model uses the Leave-One-Subject-Out Cross-Validation (LOSO) validation technique and does not resample the dataset, achieving accuracy, sensitivity, specificity, and AUROC values of 99.57%, 99.57%, 99.47%, and 99.58%, respectively.

Upon resampling the data and ensuring that each class (N, L, R, A, and V) accounts for 20% of the sample size, the metrics slightly decrease, reaching values of 98.69, 98.69, 98.38, and 98.71%, respectively. Given the imbalanced nature of the MIT-BIH dataset, this variation is unrealistic, as it affects the metrics of the less dominant classes.

If, after resampling, Leave-One-Subject-Out Cross-Validation (LOSO) is eliminated, replacing it with a random 80/20 data partition, the results are increased to 96.64, 99.64, 99.9, and 99.71%. These results are almost perfect and surpass the state of the art (see Table 8), but this variant is also unrealistic. It can introduce data from the same patient in both training and validation, artificially inflating the model’s performance due to the morphological specificity of the ECG between subjects. Therefore, it is discarded.

Tests were also run by reducing the four convolutional layers to three, and the metrics decreased slightly. Finally, the kernel sizes of the four convolutional layers (11, 13, 15, and 17) were incremented to (13, 15, 17, and 19), and the metrics continued to decrease (see Table 8).

**Figure 9:**
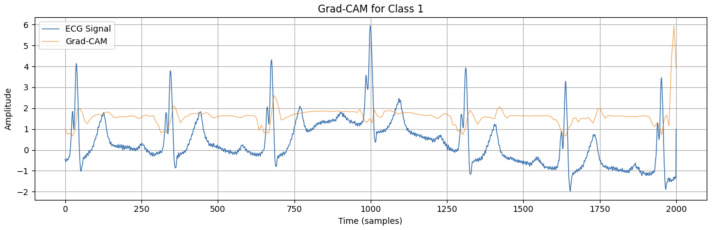
Grad-CAM in the first input sample on the MIT-BIH arrhythmia database.

The input regions of the ECG signals that were most important for our multiclass 1D CNN model to make a decision were identified by applying the Grad-CAM explanation technique to the first input sample (see Figure 9). The most important region is the R peak.

## 7. Conclusions

This study proposes a model for arrhythmia detection using smartwatch ECGs. A 1D CNN architecture was used to train and test our binary model on two datasets: the UMass Medical School Simband dataset, which was used to evaluate the effectiveness and efficiency of our binary model on smartwatch data, and the MIT-BIH arrhythmia database, which was used to compare the results of our multiclass model with the most recent state-of-the-art proposals.

A four-layer 1D CNN architecture was proposed, making the model lightweight and of low complexity and ensuring its subsequent deployment on smartwatches, as smartwatches currently have limited computational power.

The effectiveness results of the binary model on the UMass Medical School Simband dataset achieved an accuracy of 64.81%, sensitivity of 89.47%, and specificity of 6.25%. The results were acceptable. Since sensitivity is the metric that evaluates true positives and is most relevant in a clinical context, the result (89.47%) suggests high clinical reliability.

The efficiency results of the binary model reached 1.2 million parameters and 68.48 MFlops. This result demonstrates the model’s efficiency, which is also because the 1D CNN method used has lower computational complexity than state-of-the-art methods: 2D CNN, LSTM, hybrids, and Transformers.

The MIT-BIH arrhythmia database is a commonly used dataset in recent proposals for multiclass arrhythmia detection in Holter ECGs. Our multiclass model was tested on this dataset and achieved an accuracy of 99.57%, sensitivity of 99.57%, and specificity of 99.47%. These results make it a cutting-edge proposal and demonstrate that our model is also effective in detecting multiclass arrhythmias by processing electrocardiogram data from Holter devices.

## Figures and Tables

**Table 1:** A summary of the results of the different works tested on the MIT-BIH Arrhythmia dataset.

Paper	Year	Method	Accuracy (%)	Sensitivity (%)	Specificity (%)
Xia et al. [25]	2017	CNN	98.63	98.79	98.87
Ullah et al. [26]	2020	CNN	99.11	97.91	99.61
Zubair et al. [27]	2022	CNN	96.36	70.60	96.16
Rizqyawan et al. [28]	2022	DCNN	90.22	35.64	87.87
Ojha et al. [29]	2022	CNN	99.53	98.24	97.58
Jamil & Rahman [30]	2022	DCNN	94.84	100.00	99.60
Chen et al. [7]	2020	CNN + LSTM	99.32	97.50	98.70
Hassan et al. [8]	2022	CNN + BiLSTM	98.00	91.00	-
Midani et al. [31]	2023	CNN + BiLSTM	99.46	97.10	99.57
Alamatsaz et al. [9]	2024	CNN + LSTM	98.24	-	-
Akan et al. [32]	2023	Transformer-Attention	98.00	98.00	-
Islam et al. [4]	2024	Transformer-Attention	99.14	-	-
El-Ghaish et al. [6]	2024	Transformer-Attention	99.35	-	-
Kim et al. [5]	2025	Transformer-Attention	99.58	-	-
Busia et al. [33]	2024	TinyML	98.97	-	-
Kim et al. [34]	2023	TinyML	97.00	-	-

**Table 2:** A summary of the results of different works tested on smartwatch datasets.

Paper	Year	Data	Method	Patients	Accuracy (%)	Sensitivity (%)	Specificity (%)
Avran et al. [12]	2021	Smartwatch ECG Samsung Galaxy Watch 2	Machine Learning	204	-	88	97
Ploux et al. [13]	2022	Smartwatch ECG Apple Watch 4	DNN	260	92	91	94
Ford et al. [14]	2022	Smartwatch ECG Apple Watch 4	Machine Learning	125	87	68	93
Abu-Alrub et al. [15]	2022	Smartwatch ECG Samsung Galaxy Watch 3	Machine Learning	200	-	88	81
Wasserlauf et al. [16]	2023	Smartwatch ECG Apple Watch 4	CNN	250	-	25	99
Mannhart et al. [17]	2023	Smartwatch ECG Samsung Galaxy Watch 4	CNN	201	-	58	75

**Table 3:** Results in UMass Medical School Simband dataset per class.

Class	Accuracy (%)	Sensitivity (%)	Specificity (%)
(N) Normal	64.81	6.25	89.47
(A) Arrhythmia	64.81	89.47	6.25

**Table 4:** Results in MIT-BIH arrhythmia database per class.

Class	Accuracy (%)	Sensitivity (%)	Specificity (%)
N	99.62	99.77	99.19
L	99.98	99.94	99.98
R	99.98	99.94	99.98
A	99.70	93.19	99.87
V	99.86	98.99	99.93

**Table 5:** Comparison of our binary model with the state-of-the-art proposals using the UMass Medical School Simband dataset.

Paper	Year	Data	Method	Patients	Accuracy (%)	Sensitivity (%)	Specificity (%)
Avran et al. [12]	2021	Smartwatch ECG Samsung Galaxy Watch 2	Machine Learning	204	-	88	97
Ploux et al. [13]	2022	Smartwatch ECG Apple Watch 4	DNN	260	92	91	94
Ford et al. [14]	2022	Smartwatch ECG Apple Watch 4	Machine Learning	125	87	68	93
Abu-Alrub et al. [15]	2022	Smartwatch ECG Samsung Galaxy Watch 3	Machine Learning	200	-	88	81
Wasserlauf et al. [16]	2023	Smartwatch ECG Apple Watch 4	CNN	250	-	25	99
Mannhart et al. [17]	2023	Smartwatch ECG Samsung Galaxy Watch 4	CNN	201	-	58	75
Our Proposal	2025	Smartwatch ECG Samsung Simband 2	CNN	37	64.81	89.47	6.25

**Table 6:** Comparison of our multiclass model with the state-of-the-art proposals using the MIT-BIH arrhythmia database.

Paper	Year	Method	Accuracy (%)	Sensitivity (%)	Specificity (%)
Xia et al. [25]	2017	CNN	98.63	98.79	98.87
Ullah et al. [26]	2020	CNN	99.11	97.91	99.61
Zubair et al. [27]	2022	CNN	96.36	70.60	96.16
Rizqyawan et al. [28]	2022	DCNN	90.22	35.64	87.87
Ojha et al. [29]	2022	CNN	99.53	98.24	97.58
Jamil & Rahman [30]	2022	DCNN	94.84	100.00	99.60
Chen et al. [7]	2020	CNN + LSTM	99.32	97.50	98.70
Hassan et al. [8]	2022	CNN + BiLSTM	98.00	91.00	-
Midani et al. [31]	2023	CNN + BiLSTM	99.46	97.10	99.57
Alamatsaz et al. [9]	2024	CNN + LSTM	98.24	-	-
Akan et al. [32]	2023	Transformer-Attention	98.00	98.00	-
Islam et al. [4]	2024	Transformer-Attention	99.14	-	-
El-Ghaish et al. [6]	2024	Transformer-Attention	99.35	-	-
Kim et al. [5]	2025	Transformer-Attention	99.58	-	-
Busia et al. [33]	2024	TinyML	98.97	-	-
Kim et al. [34]	2023	TinyML	97.00	-	-
Our Proposal	2025	CNN	99.57	99.57	99.47

**Table 7:** Variants of the model tested on the UMass Medical School Simband dataset.

Variant	Accuracy (%)	Sensitivity (%)	Specificity (%)
With Leave-One-Subject-Out Cross-Validation (LOSO) and without resampling (proposal)	64.81	89.47	6.25
With Leave-One-Subject-Out Cross-Validation (LOSO) and with resampling	66.05	98.72	52.10
Without Leave-One-Subject-Out Cross-Validation (LOSO) and with resampling	98.73	98.73	97.52
With three-layer Conv1D	43.21	59.21	52.10
Size kernel (13, 15, 17, 19)	58.02	71.49	26.04

**Table 8:** Variants of the model tested on the MIT-BIH arrhythmia database.

Variant	Accuracy (%)	Sensitivity (%)	Specificity (%)
with Leave-One-Subject-Out cross-validation (LOSO) and without resampling (proposal)	99.57	99.57	99.47
with Leave-One-Subject-Out cross-validation (LOSO) and with resampling	98.69	98.69	98.38
without Leave-One-Subject-Out cross-validation (LOSO) and with resampling	99.64	99.64	99.90
with three-layer Conv1D	99.47	98.11	99.75
size kernel (13, 15, 17, 19)	98.69	98.17	99.77

## Data Availability

The data presented in this study are available in Synapse at https://www.synapse.org/Synapse:syn23565056/wiki/608635 (accessed on 13 July 2025), reference number [18,19]. And PhysioNet at https://www.physionet.org/content/mitdb/1.0.0/ (accessed on 13 July 2025), reference number [20].
